# Correlation Between Metabolic Score for Visceral Fat and Cardiovascular-Kidney-Metabolic Syndrome: Analysis of NHANES 2011–2020

**DOI:** 10.3390/healthcare13070694

**Published:** 2025-03-21

**Authors:** Xi Fang, Xuemin Yin, Qianfang Liu, Jing Liu, Ying Li

**Affiliations:** 1 Department of Nephrology, The Second Xiangya Hospital, Central South University, Changsha 410011, China; fangxi0321@csu.edu.cn (X.F.); 228211029@csu.edu.cn (X.Y.); 228202058@csu.edu.cn (Q.L.); 168112182@csu.edu.cn (J.L.); 2 Key Laboratory of Kidney Disease and Blood Purification in Hunan Province, Changsha 410011, China

**Keywords:** NHANES, BMI, visceral adiposity, Metabolic Score for Visceral Fat, CKM risk

## Abstract

**Background:** Cardiovascular-kidney-metabolic (CKM) syndrome with high incidence and mortality rates is a prevalent health issue globally. The Metabolic Score for Visceral Fat (METS-VF), as a new index for valuating visceral adipose tissue, has been reported to be closely related to a variety of diseases. However, whether the METS-VF can be an indicator to predict the risk of CKM syndrome remains unclear. **Methods:** We selected National Health and Nutrition Examination Survey (NHANES) database data from the 2011–2020 year cycles and conducted analyses between the METS-VF and CKM syndrome utilizing weighted Cox regression models, subgroup and interaction analysis, and restricted cubic spline (RCS) analysis. We also used receiver operating characteristic (ROC) curves to analyze and compare the diagnostic predictive ability of the METS-VF, the BMI, and other indicators assessing adipose tissue, including the VAI, fat mass, and lean mass, in CKM syndrome. **Results:** In this study, the average age was 34.40 ± 0.61 years in the non-CKM patients, while the average age was over 40.38 ± 0.62 years in the CKM patients. Additionally, there was a greater proportion of male patients in the CKM patients (over 49.04%) in comparison with the non-CKM patients (37.94%). The average METS-VF was higher in the CKM patients (over 6.63 ± 0.02) compared with the non-CKM patients (5.62 ± 0.03). We found the METS-VF had a positive correlation with CKM syndrome and was hardly affected by other confounding factors. The METS-VF was more closely associated with CKM syndrome in the subgroup of age 20–59 and female patients. In addition, the METS-VF had better diagnostic ability for CKM syndrome than the body mass index (BMI) and other indicators. **Conclusions:** The METS-VF is a potentially actionable indicator that had a positive correlation with CKM risk. The METS-VF may be used as a possible reference in the management of CKM syndrome.

## 1. Introduction

In recent years, a new disease syndrome has emerged, including cardiovascular disease (CVD); chronic kidney disease (CKD); and metabolic diseases such as diabetes mellitus (DM), obesity, and hypertension. High-salt and -fat diets, low physical activity, and high blood pressure are lifestyle-related risk factors for these diseases. These diseases have certain common pathophysiology mechanisms. Therefore, in 2023, the American Heart Association (AHA) named this syndrome cardiovascular-kidney-metabolic (CKM) syndrome [1]. CKM syndrome is a multisystem syndrome that can lead to multiple-organ dysfunction and frequent cardiovascular events. CKM syndrome has been categorized into five stages, ranging from no risk factors (stage 0) to severe multisystem dysfunction (stage 4), representing a progression of cardiovascular, kidney, and metabolic risks [2]. Multiple studies have shown that patients with cardiovascular disease combined with kidney disease and metabolic diseases such as obesity and diabetes significantly increased their mortality. The prevalence of these diseases alone is high, and the prevalence of multiple diseases at the same time is also high [3,4,5,6,7]. Recently, an article on the prevalence of CKM syndrome in the American population was published in the Circulation journal. That article showed that CKM syndrome affected more than 25% of the U.S. population [2]. Therefore, this syndrome, with high incidence and mortality rates globally, is a prevalent health issue, and the disease burden it brings to the global community is increasing [8]. Thus, it is of great significance to find effective measures to prevent and treat CKM syndrome.

Fat accumulation is the main pathological mechanism of CKM syndrome [1]. Increasing numbers of studies have shown that metabolic disorders based on visceral adipose tissue are associated with the risk of cardiometabolic disease [9,10]. Magnetic resonance imaging (MRI) is the gold standard for visceral adipose tissue, but its application is limited due to the high operating costs and need for highly trained personnel [11]. The body mass index (BMI) has been an important indicator for assessing obesity (BMI above 30 kg/m^2^). Obesity is a global health problem and defined as the excessive accumulation or abnormal distribution of body fat [12,13,14]. Although previous studies have shown that the BMI is an important indicator for assessing fat, there are still some limitations. For example, the BMI, which only measures body weight in relation to height, cannot reflect the distribution of fat in the body and is limited by the inability to identify individuals with high metabolic risk [15,16,17,18]. In recent years, the Metabolic Score for Visceral Fat (METS-VF), as a new index for valuating visceral adipose tissue, has been reported to be closely related to a variety of diseases, which better assists the diagnosis and treatment of diseases [19,20]. Unlike traditional adiposity markers, such as MRI and the BMI, the METS-VF incorporates factors like age, sex, the metabolic score for insulin resistance index (METS-IR), and the waist-to-height ratio (WHtR), making it more effective at reflecting visceral fat distribution and predicting associated risks. Recent studies found that the METS-VF had a better ability to assess visceral adipose and reflected the distribution of fat in human organs and tissues, which more directly indicated the occurrence of diseases [21,22,23]. The METS-VF was also found to be closely associated with renal and cardiac diseases in many studies [24,25]. However, the correlation between the METS-VF and CKM syndrome has not been studied.

Therefore, we propose a hypothesis: the METS-VF may have a positive correlation with CKM risk. The objective of this study was to investigate whether the METS-VF is significantly correlated with CKM syndrome and whether it can serve as a potentially actionable indicator for the early diagnosis and effective management of CKM. We selected National Health and Nutrition Examination Survey (NHANES) database data to conduct our study. The NHANES database is an important epidemiological survey focused on gathering information from a representative sample of participants. It collects data on health and nutrition. The information enables analysis of diet, chronic disease prevalence, lifestyle, and environmental factors among Americans.

## 2. Materials and Methods

### 2.1. Research Design and Population Selection

**Figure 1:**
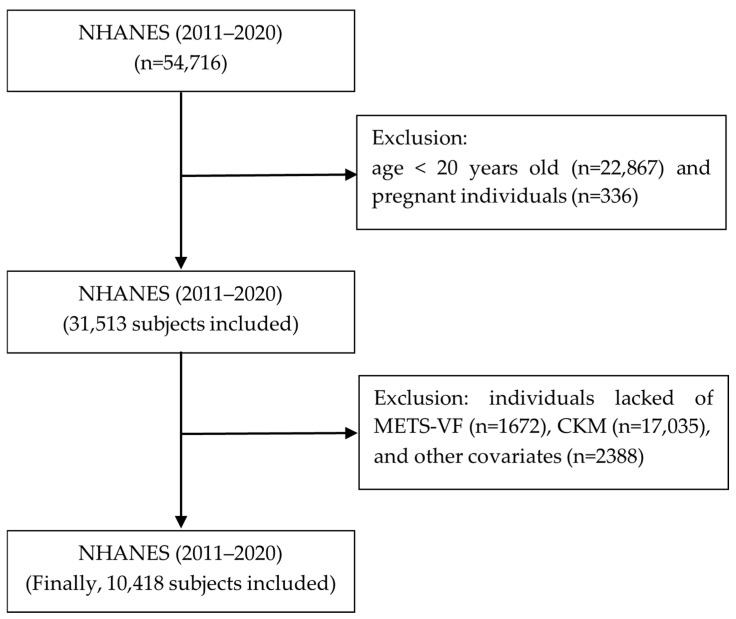
Flowchart of the individuals included.

We conducted a cross-sectional study by selecting NHANES database data from the 2011–2020 year cycles; filtering individuals aged ≥20 years old; and removing pregnant individuals, as well as individuals with missing METS-VF and CKM data, as well as other covariates. The total number of individuals was 54,716, and after removing the individuals aged <20 years old (n = 22,867) and pregnant individuals (n = 336), 31,513 individuals remained. After removing the individuals with missing data on the METS-VF (n = 1672) and CKM syndrome (n = 17,035) as well as other covariates (n = 2388), 10,418 individuals remained (Figure 1).

### 2.2. Observed Variable

#### 2.2.1. Definition of the METS-VF Index and CKM Syndrome

The METS-VF was used to assess the distribution of visceral fat. The higher the METS-VF, the higher the fat content of the organ or tissue. The METS-VF combined several factors, including the metabolic score for insulin resistance index (METS-IR), the waist-to-height ratio (WHtR), sex, and age.

CKM syndrome was divided into 5 stages: 0 (no associated risk factors); 1 (obesity); 2 (diabetes mellitus or hypertension or chronic kidney disease with medium–high risk); 3 (very high risk of chronic kidney disease or predicted risk of CVD within 10 years); and 4 (suffering from CVD). Staging was based on BMI, waist circumference (WC), DM, CKD, and CVD [2]. All variables, including anthropometric and metabolic data, were obtained from the NHANES database from 2011 to 2020 through interviews, clinical measurements, and laboratory testing. The detailed protocol of the data collection is described at http://www.cdc.gov/nchs/nhanes (accessed on 20 October 2024).

The definition of the METS-VF, METS-IR, and WHtR were determined by the following formula:

METS-VF = 4.466 + 0.011 [(Ln (METS-IR))^3^] + 3.239 [(Ln (WHtR))^3^] + 0.319 (Sex) + 0.594 (Ln (Age))

METS-IR = Ln [(2 × fasting glucose) + fasting triglycerides × BMI]/[Ln (high-density lipoprotein cholesterol)]

WHtR = waist/height

#### 2.2.2. Covariate Selection

All covariates were also obtained from the NHANES database from 2011 to 2020. A number of demographic factors were selected in this study, such as age, sex, and race, as well as smoking status, including never smoked, former smoker, or current smoker. Alcohol consumption was also included, assessing both non-drinkers and severity of drinking. Additionally, body measurements were gathered, focusing on the BMI, fat mass, lean mass, the visceral adiposity index (VAI), and systolic blood pressure (SBP). Laboratory tests were gathered including fasting glucose, fasting triglycerides, and high-density lipoprotein cholesterol. Comorbidities included hypertension, indicated by blood pressure readings over 140/90 mmHg, and diabetes, defined by HbA1c of 6.5% or higher or fasting blood glucose of 7.0 mmol/l or more. Diabetes may also have been indicated by the use of diabetes medications or insulin. CKD was classified into five stages with eGFR according to the KDIGO guidelines [26]. Additionally, cardiovascular diseases included heart attack, stroke, coronary heart disease, and congestive heart failure.

The definition of the BMI and VAI were determined by the following formula:

BMI = weight (kg)/height^2^ (m^2^)

VAI (male) = (WC/(39.68 + 1.88 × BMI)) × (TG/1.03) × (1.31/HDL); VAI (female) = (WC/(36.58 + 1.89 × BMI)) × (TG/0.81) × (1.52/HDL)

### 2.3. Statistical Analysis

The NHANES database was utilized to represent the population with weighting, employing various statistical analysis. Means ± standard error (SE) were used to represent numerical quantitative variables, while percentages (%) were used to represent character qualitative variables. Specific analysis methods included, for comparisons in groups, weighted *t*-tests, one-way analysis of variance, and chi-squared tests. Bonferroni’s correction was conducted for multiple comparisons to reduce the likelihood of false positives. To identify the correlation between the METS-VF and CKM syndrome across different models, multivariable Cox regression analysis, collinearity diagnostics analysis, and restricted cubic spline (RCS) regression were performed. To clarify the diagnostic performance of the METS-VF, receiver operating characteristic (ROC) curves were used.

*p* < 0.05 represented a statistically significant difference. R 4.4.2 software was used to process the data and perform various analyses.

## 3. Results

### 3.1. Characteristics of the Baseline Population

As shown in Table 1, with the increase in the CKM stage, the age increased gradually. The average age was 34.40 ± 0.61 years in the CKM 0 patients, while the average age was over 40.38 ± 0.62 years in the CKM 1–4 patients. Additionally, there was a greater proportion of male patients in the CKM 1–4 patients (over 49.04%) in comparison with the CKM 0 patients (37.94%). In the CKM patients, the majority of the affected races were Non-Hispanic White, accounting for more than 63.44%. Most patients had alcohol consumption in the CKM group (over 64.59%). The CKM 1–4 patients had average BMIs of over 28.30 ± 0.17, which was higher than the CKM 0 patients (21.71 ± 0.10). The average fat mass was higher in the CKM 1–4 patients (over 28.30 ± 0.17) compared with the CKM 0 patients (17.42 ± 0.25); however, the opposite result existed for lean mass—the average VAI was higher in the CKM 1–4 patients (over 1.01 ± 0.02) compared with the CKM 0 patients (0.77 ± 0.02), and the average METS-VF was higher in the CKM 1–4 patients (over 6.63 ± 0.02) compared with the CKM 0 patients (5.62 ± 0.03). All the differences mentioned above are statistically significant (*p* < 0.0001).

### 3.2. The Correlation Between the METS-VF and CKM Syndrome

To investigate whether the METS-VF correlates with CKM syndrome, we obtained follow-up data from the NHANES database and conducted a weighted Cox regression analysis. The analysis, as shown in Table 2, revealed significant correlations between the METS-VF and CKM syndrome. Specifically, we found that the METS-VF had a positive correlation with CKM syndrome across three models—model 1 (HR = 1.377; 95% CI 1.310, 1.448; *p* < 0.0001), model 2 (HR = 1.363; 95% CI 1.266, 1.468; *p* < 0.0001), and model 3 (HR = 1.665; 95% CI 1.457, 1.903; *p* < 0.0001)—with increasing METS-VFs corresponding to higher incidences of CKM syndrome. In the tertile comparisons shown in model 1, the highest METS-VF values (Tertile 3) showed the highest CKM syndrome incidence (HR = 1.477; 95% CI 1.373, 1.589; *p* < 0.0001), while the lower the METS-VF value (Tertile 2), the lower the incidence of CKM syndrome (HR = 1.438; 95% CI 1.318, 1.570; *p* < 0.0001). In the tertile comparisons shown in model 2, the highest METS-VF values (Tertile 3) showed the highest CKM syndrome incidences (HR =1.359; 95% CI 1.228, 1.503; *p* < 0.0001), while the lower the METS-VF value (Tertile 2), the lower the incidence of CKM syndrome (HR = 1.340; 95% CI 1.186, 1.514; *p* < 0.0001). In the tertile comparisons shown in model 3, the higher METS-VF values (Tertile 2) showed higher CKM syndrome incidence (HR = 1.153; 95% CI 1.031, 1.289; *p* < 0.009), but no significant correlation (*p* = 0.967) was seen in Tertile 3.

### 3.3. Collinearity Diagnostics Analysis

To assess the robustness of the METS-VF as a standalone marker, we conducted collinearity diagnostics analysis. As shown in Table 3, the results showed that the variance inflation factor (VIF) values were all less than 2, which indicated that there was no multicollinearity for the indicators including BMI, VAI, fat mass, and lean mass. The results suggest that the METS-VF is an independent indicator.

### 3.4. Subgroup and Interaction Analysis

**Figure 2:**
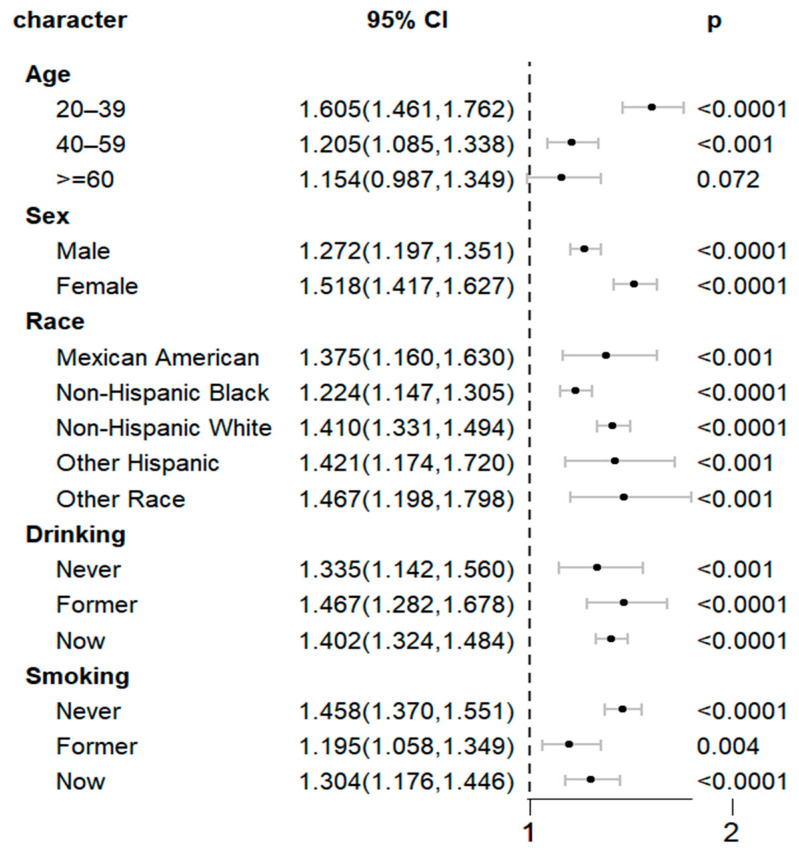
Subgroup analysis of METS-VF and CKM syndrome using weighted Cox regression analysis.

To further study the relationship between the METS-VF and CKM syndrome in different subgroups of the population, Table 4 shows that when the METS-VF was divided into three intervals according to numerical value, specifically, Tertile 3 was in the highest METS-VF category. The METS-VF was most closely associated with CKM syndrome in the subgroup of age 20–59. The METS-VF was more positively associated with CKM syndrome in female patients. The correlation between the METS-VF and CKM syndrome also varied among different ethnic groups. There was a more significant positive correlation between the METS-VF and CKM syndrome in the drinking population. Interaction analysis showed no significant correlation between the METS-VF and other factors, indicating that the METS-VF is an independent factor to predict CKM syndrome. We used a forest plot to analyze the subgroup analysis between the continuous variables for the METS-VF and CKM syndrome and found that the METS-VF was most closely associated with CKM syndrome in the subgroups of age 20–59, female, and other-race patients. (Figure 2).

### 3.5. RCS Analysis

**Figure 3:**
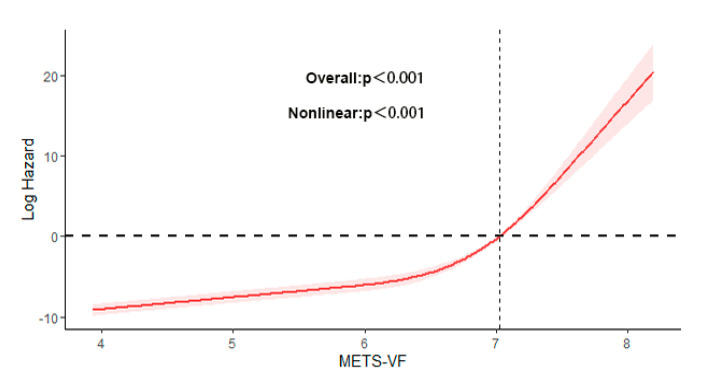
Restricted cubic spline regression analysis. Non-linear relationship between METS-VF and CKM syndrome. Adjusted for factors including age, sex, race, smoking, drinking, systolic blood pressure, BMI, fat mass, lean mass, VAI, and hypertension.

To further visually observe the impact of the METS-VF on the development of CKM syndrome, we conducted restricted cubic spline regression analysis and found that when the METS-VF was <7.023, as the METS-VF increased, the risk of suffering CKM syndrome increased at a slower rate. When the METS-VF was >7.023, as the METS-VF increased, the risk of suffering CKM syndrome increased at an accelerated rate, which indicated that there was a non-linear relationship between the METS-VF and CKM syndrome (Figure 3).

### 3.6. ROC Analysis

**Figure 4:**
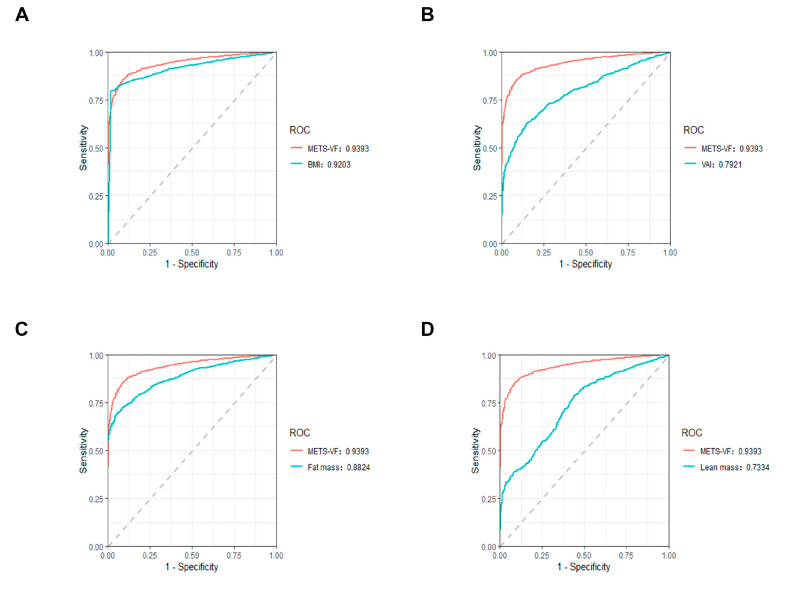
ROC analysis of METS-VF and other indices in the diagnosis of CKM syndrome. ROC curves between (**A**) METS-VF, BMI, and CKM syndrome; (**B**) METS-VF, VAI, and CKM syndrome; (**C**) METS-VF, fat mass, and CKM syndrome; and (**D**) METS-VF, lean mass, and CKM syndrome.

Next, ROC analysis was conducted on the METS-VF, the BMI, and other indicators related to the assessment of adipose tissue to detect their diagnostic predictive significance in CKM syndrome. The findings showed that the METS-VF and BMI had high AUC values for CKM syndrome and had good predictive power. The predictive ability of the METS-VF (AUC: 0.9393) for CKM syndrome was better than the BMI (AUC: 0.9203) and other indicators assessing adipose tissue, including the VAI, fat mass, and lean mass (Figure 4).

## 4. Discussion

In this large-data study, we showed that the METS-VF is positively associated with CKM risk. Firstly, we found that compared with non-CKM patients, CKM patients had increased METS-VF levels. Then, through different weighted regression model analyses, RCS, ROC, and subgroup and interaction analysis, we found that the METS-VF had a positive correlation with CKM syndrome and was hardly affected by other confounding factors; the METS-VF had better diagnostic ability for CKM syndrome than the BMI and other indicators assessing adipose tissue, including the VAI, fat mass, and lean mass.

In the process of clinical diagnosis and treatment, we have often observed that a patient suffers from a variety of diseases at the same time: the more common are cardiovascular, kidney, and metabolic diseases; these three diseases often affect each other; and the morbidity and mortality of these diseases are very high, which brings a heavy burden to the public health system [1,27]. Therefore, the emergence of CKM syndrome has provided more comprehensive diagnosis and treatment for these patients. In recent years, clinical and basic research has led to a growing understanding of the complex interactions between metabolic factors, CKD, and CVD [28,29,30,31]. Excessive accumulation or loss of function of fat is the most common pathophysiological mechanism of CKM syndrome. Adipose tissue can be distributed in various parts of the human body, including subcutaneous tissue and internal organs, etc. When there is too much adipose tissue in the internal organs, hypertrophic adipocytes and tissue-resident immune cells experience phenotype changes that produce a series of pro-inflammatory and pro-oxidative cytokines and chemokines, both locally and systemically, to induce peripheral insulin resistance, which can damage the blood vessels, kidneys, and heart and eventually cause damage to multiple organs [32,33,34]. Additionally, recent studies have shown that ectopic fat, such as hepatic and pericardial fat, is also associated with cardiometabolic disease [35,36]. These findings suggest that there is a link between visceral fat, ectopic fat, and CKM risk. The METS-VF has been a metabolic indicator of visceral fat accumulation and also associated with ectopic fat [35,37,38]. Therefore, the METS-VF is a potentially predictable indicator of CKM risk. In addition, previous research has indicated that the METS-VF is closely related to cardiac or metabolic disease [24,25,39,40,41]. The METS-VF has been associated with an increased risk of CVD and could be used as a predictive index of the risk of CVD [24,40,41]. The METS-VF may be an indicator for predicting CKD risk [25]. The METS-VF could also assess cardiometabolic risk [20]. On the basis of the above research, our study found that there was a positive relationship between the METS-VF and CKM risk. Since our study was a cross-sectional study, prospective clinical studies are needed in the future.

The METS-VF reflects the amount and distribution of fat, while the BMI, which represents body weight, does not directly reflect the distribution of fat. The BMI is often used to assess weight and is widely used as a simple and easily accessible indicator. Precisely because it is too simple, it has many limitations in its specific application, as it does not reflect overall health status [35,42,43]. Therefore, from this point of view, the METS-VF is a better predictor of CKM syndrome than the BMI. In our study, we also found that the ability of the METS-VF to predict CKM risk was stronger than that of the BMI, which is consistent with previous studies. In one study, the METS-VF was similarly found to be superior to indicators such as the BMI in predicting CKD risk [25]. In another study, to predict the risk of hypertension, researchers compared the METS-VF to other predictors of fat and found that the METS-VF was superior to the BMI [44]. In the assessment of some cardiovascular or metabolic diseases caused by fat accumulation, many studies have considered the METS-VF to be superior to the BMI [14,45]. Other composite indicators commonly used to assess visceral fat in most studies are the VAI, the WHtR, etc. The METS-VF significantly identifies diabetes compared to other visceral fat indices, such as the VAI and WHtR [45,46]. Similar results were seen in CKD [25]. MRI can accurately assess visceral fat, but its use is limited by its high cost, time consumption, and limited availability. One study has shown that the METS-VF showed better performance compared to MRI [20]. More research is needed to further prove this, but it still hints that the METS-VF is a very valuable indicator. The METS-VF, as a comprehensive indicator of multiple parameters (sex, age, blood glucose, lipoprotein, and WHtR), has been applied to various diseases [21,47,48]. Consistently with the above research, our study found that the METS-VF had a better predictive ability for the diagnosis of CKM syndrome than the VAI, fat mass, and lean mass. Based on these, we believe that the METS-VF is a credible index that is related to CKM syndrome.

There have been some studies on the threshold of the METS-VF in cardiac or metabolic disease. In one study, the optimal cut-off value of the METS-VF associated with CVD incidence was ≥6.80 [49]. In another study, stroke risk showed a sharp rise when the METS-VF exceeded 7.00 [50]. In our study, we found that when the METS-VF was >7.023, the correlation between the METS-VF and CKM syndrome was stronger, which suggests that when the METS-VF is >7.023, the risk of CKM syndrome is greater. Our results are basically consistent with previous studies. In our study, subgroup analysis showed that the METS-VF was most closely associated with CKM syndrome in the subgroup of age 20–59, female, and other-race patients. This result is consistent with the results of several recent studies on cardiac and metabolic diseases [40,46,51]. This may be related to the fact that older people are less overnourished and men have less fat than women.

This study utilized the NHANES database, which was a study with large and long-term follow-up data along with good reliability. Our study also weighted the data to better represent the overall population and had good representativeness. We also controlled multiple confounding factors and used multiple analytical methods to ensure credible results. However, this study also acknowledges certain limitations. It did not include all confounding variables. Some major variables were mainly analyzed in our study, so more covariates, such as lifestyle factors, need to be included in future studies. In addition, the investigated data of this study were from the USA. Therefore, the sample size of future studies can also be extended to other countries and different populations. More future research is needed to compare the METS-VF to various current models predicting cardiometabolic risk by calibration and reclassification analyses. Large clinical prospective studies are also necessary.

## 5. Conclusions

The METS-VF is a potentially actionable indicator that had a positive correlation with CKM risk. The METS-VF may be used as possible reference in the management of CKM syndrome, but more future prospective clinical studies with robust designs are still needed.

## Figures and Tables

**Table 1:** Weighted characteristics of the baseline population.

Characteristics	CKM 0	CKM 1	CKM 2	CKM 3	CKM 4	*p*-Value
	(n = 838)	(n = 2138)	(n = 5797)	(n = 538)	(n = 1107)	
Age	34.40 (0.61)	40.38 (0.62)	48.73 (0.34)	74.93 (0.46)	64.52 (0.56)	<0.0001
Sex						<0.0001
Female	62.06 (2.22)	50.96 (1.60)	48.12 (0.86)	36.99 (3.12)	42.81 (2.28)	
Male	37.94 (2.22)	49.04 (1.60)	51.88 (0.86)	63.01 (3.12)	57.19 (2.28)	
Race						<0.0001
Mexican American	4.72 (0.74)	10.72 (1.40)	9.35 (0.80)	4.78 (0.90)	3.99 (0.69)	
Non-Hispanic Black	7.11 (0.80)	9.46 (0.86)	10.50 (0.94)	12.15 (1.55)	10.53 (1.08)	
Non-Hispanic White	73.62 (2.03)	63.44 (2.04)	65.87 (1.58)	68.93 (2.79)	74.05 (1.94)	
Other Hispanic	5.87 (1.13)	7.48 (0.74)	5.92 (0.61)	6.42 (1.24)	4.10 (0.65)	
Other Race	8.69 (1.07)	8.90 (0.85)	8.37 (0.50)	7.72 (1.35)	7.32 (1.17)	
Drinking						<0.0001
Never	9.57 (1.50)	8.48 (0.97)	9.65 (0.58)	17.62 (2.39)	11.18 (1.13)	
Former	4.12 (0.91)	5.17 (0.53)	9.00 (0.62)	17.78 (2.30)	20.42 (1.83)	
Now	86.32 (1.85)	86.35 (1.20)	81.35 (0.88)	64.59 (2.81)	68.40 (1.99)	
Smoking						<0.0001
Never	68.68 (2.17)	60.21 (1.43)	55.30 (1.02)	45.29 (3.37)	38.49 (2.11)	
Former	15.08 (1.66)	24.20 (1.61)	25.49 (0.92)	39.46 (3.60)	39.15 (2.16)	
Now	16.24 (1.49)	15.59 (1.22)	19.20 (0.78)	15.26 (1.94)	22.36 (2.33)	
SBP	108.12 (0.42)	112.25 (0.29)	125.53 (0.28)	144.39 (1.52)	128.62 (0.78)	<0.0001
Hypertension						<0.0001
No	100.00 (0.00)	100.00 (0.00)	46.23 (1.00)	19.70 (2.55)	27.66 (2.12)	
Yes	0.00 (0.00)	0.00 (0.00)	53.77 (1.00)	80.30 (2.55)	72.34 (2.12)	
BMI	21.71 (0.10)	28.30 (0.17)	30.79 (0.17)	28.96 (0.30)	30.74 (0.33)	0.0001
Fat mass	17.42 (0.25)	27.91 (0.32)	32.46 (0.29)	29.41 (0.56)	32.42 (0.56)	< 0.0001
Lean mass	43.15 (0.39)	51.33 (0.32)	54.25 (0.26)	50.20 (0.68)	52.81 (0.60)	<0.0001
VAI	0.77 (0.02)	1.01 (0.02)	2.35 (0.05)	2.33 (0.26)	2.26 (0.10)	<0.0001
METS-VF	5.62 (0.03)	6.63 (0.02)	7.01 (0.01)	7.41 (0.02)	7.31 (0.03)	<0.0001

Note: Means ± standard error (SE) are used to represent numerical quantitative variables and percentages are used to represent character qualitative variables.

**Table 2:** Correlation analysis between METS-VF and CKM syndrome.

	Model 1		Model 2		Model 3	
	HR (95%CI)	*p*-Value	HR (95%CI)	*p*-Value	HR (95%CI)	*p*-Value
METS-VF						
METS-VF as continuous variable	1.377 (1.310, 1.448)	<0.0001	1.363 (1.266, 1.468)	<0.0001	1.665 (1.457, 1.903)	<0.0001
Tertile 1	Reference		Reference		Reference	
Tertile 2	1.438 (1.318, 1.570)	<0.0001	1.340 (1.186, 1.514)	<0.0001	1.153 (1.031, 1.289)	0.009
Tertile 3	1.477 (1.373, 1.589)	<0.0001	1.359 (1.228, 1.503)	<0.0001	1.004 (0.847, 1.189)	0.967
*p* for trend		<0.0001		<0.0001		0.943

Note: This describes the transformation of continuous variables into categorical ones through tertiles and details three models for adjustment: unadjusted for Model 1; adjusted for age, sex, and race for Model 2; and further adjusted for smoking, drinking, and systolic blood pressure for Model 3.

**Table 3:** Collinearity diagnostics analysis.

Covariates	VIF	Tolerance
BMI	1.92	0.52
VAI	1.43	0.70
Fat mass	1.07	0.93
Lean mass	1.10	0.91

**Table 4:** Subgroup and interaction analysis of METS-VF and CKM syndrome.

Character	Q1	Q2	Q3	*p* for Trend	*p* for Interaction
Age					0.076
20–39	1	1.630 (1.410, 1.885)	1.478 (1.198, 1.825)	<0.0001	
40–59	1	1.262 (1.105, 1.440)	1.271 (1.109, 1.457)	0.001	
≥60	1	1.158 (0.850, 1.578)	1.248 (0.966, 1.612)	0.037	
Sex					0.07
Male	1	1.322 (1.179, 1.483)	1.403 (1.277, 1.542)	<0.0001	
Female	1	1.550 (1.400, 1.717)	1.543 (1.373, 1.735)	<0.0001	
Race					0.261
Mexican American	1	1.330 (1.079, 1.640)	1.520 (1.222, 1.891)	<0.001	
Non-Hispanic Black	1	1.214 (1.069, 1.378)	1.209 (1.086, 1.346)	<0.001	
Non-Hispanic White	1	1.466 (1.307, 1.644)	1.540 (1.403, 1.690)	<0.0001	
Other Hispanic	1	1.515 (1.222, 1.879)	1.547 (1.183, 2.022)	0.001	
Other Race	1	1.689 (1.389, 2.055)	1.401 (0.967, 2.029)	0.016	
Drinking					0.484
Never	1	1.301 (0.977, 1.733)	1.261 (0.973, 1.636)	0.095	
Former	1	1.589 (1.263, 1.998)	1.769 (1.519,2.061)	<0.0001	
Now	1	1.452 (1.309, 1.611)	1.521 (1.382, 1.674)	<0.0001	
Smoking					0.449
Never	1	1.493 (1.332, 1.673)	1.500 (1.362, 1.652)	<0.0001	
Former	1	1.277 (1.084, 1.503)	1.375 (1.183, 1.600)	<0.0001	
Now	1	1.402 (1.193, 1.647)	1.348 (1.120, 1.623)	<0.001	

Note: This uses tertiles to categorize continuous variables.

## Data Availability

Publicly available datasets were analyzed in this study. These data can be found at http://www.cdc.gov/nchs/nhanes (accessed on 20 October 2024).

## Abbreviations

The following abbreviations are used in this manuscript:

CKM	Cardiovascular-kidney-metabolic syndrome
METS-VF	Metabolic Score for Visceral Fat
NHANES	National Health and Nutrition Examination Survey
RCS	Restricted cubic spline
ROC	Receiver operating characteristic
CVD	Cardiovascular disease
CKD	Chronic kidney disease
DM	Diabetes mellitus
BMI	Body mass index
VAI	Visceral adiposity index
HR	Hazard ratio
95% CI	95% confidence interval
VIF	Variance inflation factor
